# Mitochondrial genome of the *Podosphaera xanthii*: a plant pathogen causes powdery mildew in cucurbits

**DOI:** 10.1080/23802359.2019.1618209

**Published:** 2019-11-20

**Authors:** Seunghwan Kim, Myunghee Jung, Eun A. Oh, Tae Ho Kim, Jeong-Gu Kim

**Affiliations:** a Genomics Division, National Institute of Agricultural Science, RDA, Jeonju-si, Republic of Korea;; b Research and Development Center, Insilicogen Inc, Yongin-si, Gyeonggi-do, Republic of Korea

**Keywords:** Mitochondrial genome, *Podosphaera xanthii*, plant pathogen, powdery mildew

## Abstract

In this study, we sequenced the complete mitochondrial genome of the *Podosphaera xanthii*, which is the powdery mildew diseases causative pathogen for cucurbits. The total size of the mitochondrial genome is 26,052** **bp, which includes 15 coding genes, 25 tRNAs, and 2 rRNAs. The cytochrome c oxidase subunit I (COXI) used for the phylogenetic construction, which grouped this species into *Hypocreomycetidae* taxonomy family, which could aid the researchers to place the fungal in an appropriate taxonomy clade.

*Podosphaera xanthii* is the plant pathogen belonging to *Erysiphaceae* family, and the fungal belonging to this family cause the powdery mildew disease in most cultivated plants/crops, particularly, *P. xanthii* is the pathogen for cucurbits (Martínez-Cruz et al. 2018). In Korea, this fungus effects various crops in cucurbits, such as melon (Hong et al. 2018), cucumber (Nie et al. 2015), and other weed-like plants, such as *Cirsium japonicum* (Lee 2012). This powdery mildew pathogens have specific molecular mechanism called haustoria, which is used to invade the host cells (Lee 2012; Hong et al. 2018). This fungus mostly cause serious economic loss in greenhouse environment, which decide the yield of the most cucurbits (Nie et al. 2015).

In this study, we sequenced the complete mitochondrial genome of *P. xanthii* (Submitted to Genbank with the accession number: MK674497). The samples were collected from the cucumber leaves (*Cucumis sativus*) grown in the field located in South Korea (N35°90′, E127°15′/Wanju, Jeollabuk-do) in the month of June 2018, by National Institute of Agriculture Science. The complete DNA was isolated from the fungal isolates and sequenced using the PacBio protocol, as per the given instructions. The sequencing procedures were conducted by DNALink (http://www.dnalink.com), an authorized sequence service provider in South Korea. The 26,052 bp long complete mitochondrial genome was assembled from 30.9 giga base (Gb) of whole genomic DNA sequence. The assembled mitochondrial genome was obtained and the structural annotations were done using the MITOS2 computational method, with the reference of 81 fungal data set (http://mitos2.bioinf.uni-leipzig.de/index.py), which resulted in 15 coding genes, 25 tRNAs, and 2 rRNAs. Among those, the ATP9 and NAD5 genes had the stop codon TAG and the remaining genes had TAA as the stop codon.

Finally, to elucidate the evolutionary relationship of the sequenced *P. xanthii* mitochondrial genome, among the close relative taxon, the phylogenetic tree was reconstructed as follows. The cytochrome oxidase subunit 1 (COX1) gene was used to reconstruct the phylogenetic tree along with 76 closely related species from two taxonomy class, i.e. *Sordariomycetes* and *Leotiomycetes*. The phylogenetic tree shown in Figure 1 grouped the sequenced *P. xanthii* in *Hypocreomycetidae*, rather than *Sordariomydetidae*, which denotes the importance of reorganizing this fungus in a specific taxon. The phylogenetic construction methodology was followed as instructed by Kim et al. (2018). Moreover, at present, as per the Genbank genome database status, this is the first mitochondrial genome for the genus *Podosphara*. This mitochondrial data could be a valuable resource for the genus *Podosphara* to examine the evolutionary relationship among the close species in this taxon.

**Figure 1.**
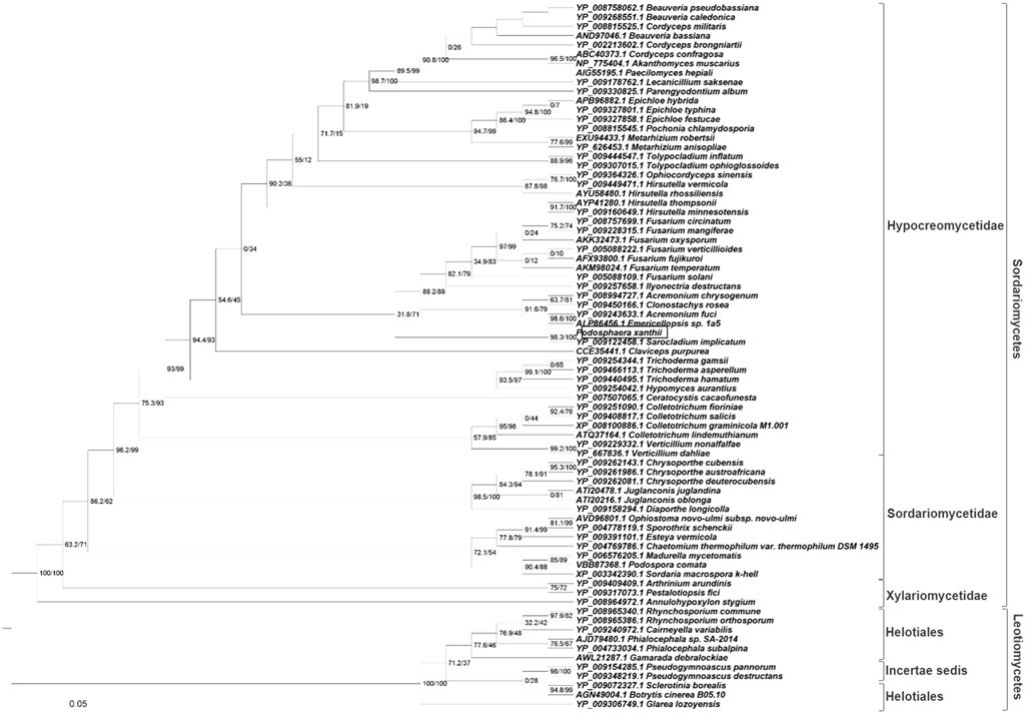
Phylogenetic tree reconstructed with seventy-six species COX1 gene from two taxonomy classes. The tree was constructed from 1000 ensemble trees generated by the neighbor-joining method. The pairwise distances between the species are presented at their respective branches.
